# “If only he were blind”: Shame, trauma, and dissociation among women with body dysmorphic disorder in physically intimate relationships

**DOI:** 10.1080/17482631.2021.2015871

**Published:** 2022-01-17

**Authors:** Natalie Stechler, Isabel Henton

**Affiliations:** Department of Counselling Psychology, Regent's University London, Inner Circle, Regent's Park, London, NW1 4NS

**Keywords:** Body dysmorphic disorder, physical intimacy, shame, trauma, dissociation, interpretative phenomenological analysis, compassion, mindfulness

## Abstract

**Background:**

Body Dysmorphic Disorder (BDD) involves a debilitating preoccupation with one’s appearance and associated difficulties in social and interpersonal relationships, according to the Diagnostic and Statistical Manual for Mental Disorders (DSM-5). Quantitative research has investigated the severity of relationship difficulties in BDD, while qualitative research has primarily focused on intrapersonal phenomena, although interpersonal difficulties, including with physical intimacy, have frequently emerged from these studies.

**Aims:**

This study explores how women with BDD make sense of their lived experiences of physical intimacy in the context of current partner relationships.

**Method:**

Six adult women participated in individual semi-structured interviews. The data was analysed using Interpretative Phenomenological Analysis.

**Results:**

The analysis generated three superordinate themes: 1) The shame in being seen, 2) Disgust and detachment during intimacy, and 3) A flawed self, unworthy of relationships.

**Conclusions:**

This study demonstrates how appearance-related concerns filter into the cognitive, behavioural, and emotional intersubjective spaces of physically intimate partnerships. Shame or trauma may be triggered and may be managed through disengagement or dissociation.

**Clinical implications:**

These findings support calls for a full psychological assessment of the contextual and interpersonal components of BDD, and further suggest that psychological interventions for shame, trauma, and dissociation, such as compassion-focused therapy, imagery rescripting, or body-focused therapies, may be helpful additions to cognitive-behavioural or exposure and response prevention interventions for practitioners working with BDD.

Body dysmorphic disorder (BDD) was first described in 1891 as “dysmorphophobia,” a fear of imagined ugliness or deformity, by Italian Psychiatrist Enrico Morselli (Morselli, 2001). It has subsequently been characterized as a chronic, debilitating experience, grounded in a preoccupation and fixation with aspect/s of one’s own appearance. According to the Diagnostic and Statistical Manual for Mental Disorders (DSM-5, American Psychiatric Association, 2013), BDD involves: (1) preoccupation with perceived flaw(s) or defect(s) that appear unnoticeable or only slightly noticeable to others, (2) engagement with repetitive behaviours or mental acts such as hypervigilant attention, mirror-checking, touching of the perceived defect, or using clothes to camouflage or hide the defect, (3) clinically significant distress and/or impairment with social or daily functioning. In psychiatry, BDD is often diagnosed through the structured clinical interview (e.g., SCID-5-CV, First et al., 2016) and is generally held to be an underdiagnosed condition. Within the general population, the prevalence of BDD ranges from 0.5 to 3.2%, with a higher prevalence among dermatology (4.9–21.1%) and cosmetic surgery cohorts (2.9–57%) (Minty & Minty, 2021).

In its original definition, the contextual role of others is central to BDD phenomenology - the ugliness or deformity is perceived by the sufferer to be in the imagination or mind’s eye of the other person. The DSM-5 adopts the perspective of the other person as an observer, to whom flaws appear either unnoticeable or only slightly noticeable. The DSM-5 diagnosis also highlights consequent social impairments, which may include interpersonal relationships at work and at home, with friends, family, and partners.

Theoretical perspectives also cast BDD as a relational, interpersonal phenomenon, and provide explanatory narratives for how BDD might come about or be experienced as so distressing for individuals. From a psychoanalytic object relations perspective, being seen or watched by the Other exposes individuals preoccupied with their appearance to the gaze of the Other in the self-other relation, which elicits anxiety. In the context of partner relationships, the individual makes sense of their body-self through the face of the desired object (in this case, the partner), and is vulnerable to a negative influence, through this lens, on their own perception of their body image (Lemma, 2009).

Phenomenological perspectives on BDD also reference the self-other relation. We exist within a body, and, through it, we experience the world (Merleau-Ponty, 1962/2002). The body oscillates between the subjective body (the lived body or “body-subject”) and the objective body (the body that I have, or “body-object” observed by others). The body-subject pre-reflectively experiences being in the world, while the body-object is purely physiological, and can be not only observed, but critiqued or changed by others. When the body-subject is seen by the Other, it becomes an object (Fuchs, 2002). From this perspective, BDD can be conceived as a tension between the body-subject and the body-object; imagining the body from the latter perspective may generate a disembodied sense of self for those with BDD (Mitchell, 2017; Morris, 2003).

Similarly, in existential phenomenology, in being seen by others, the lived-body no longer exists in and of itself but, rather, as the body-for-others (Groys, 2012; Morris, 2003; Sartre, 1969). Morris (2003) has suggested that Sartre’s reference to invisibility is relevant to the experience of BDD. Sartre suggested that dislike of one’s body manifests in a wish to be invisible and “not to have a body anymore” (Sartre, 1969, p. 353). The body is invisible until the presence of the gaze makes it visible—under someone else’s gaze, the lived-body becomes an object visible to others (body-for-others) and subject to the other person’s existence. This gaze objectifies the lived body and prevents us from being in the present moment. In a similar way, individuals living with BDD often express a longing not to be seen, and engage in hiding, concealing, or camouflaging, perhaps in an attempt to become invisible to others (Morris, 2003).

Furthermore, Fuchs (2002) and Morris (2003) suggest that being seen by others involves shame, and that shame is a central phenomenon in BDD—through being looked at, shame in one’s appearance or body is evoked. For the person with BDD, the other person’s gaze restricts the possibility of being in a body and produces a felt need to put on a performance, or adopt a role or persona. Hiding the body is conceived as an attempt to conceal a true self that is felt to be flawed. The possibility of spontaneous bodily existence is hijacked under the gaze of the other; the person with BDD is overwhelmed in being the object of the other person’s attention (Fuchs, 2002).

## Quantitative research

Quantitative research has further explored the nature of interpersonal difficulties in BDD. For example, Didie et al. (2012) investigated the severity, domains, and correlates of interpersonal problems among a US community sample of 51 individuals with BDD. Individuals with BDD reported greater severity of interpersonal problems across most domains in the Inventory of Interpersonal Problems (IIP-64, Horowitz et al., 2000) than community benchmarks. In particular, they scored significantly higher than benchmarks in social inhibition and non-assertiveness.

In line with these findings regarding social inhibition and non-assertiveness, Fang et al. (2011) found that sensitivity to rejection by others was a mediating factor in the relationship between social anxiety and body dysmorphic concerns, and that rejection sensitivity was particularly linked with the cognitive symptoms of BDD. Similarly, Webb et al. (2015) found that BDD symptoms among adolescents were associated with same-and cross-sex peer teasing via appearance-based rejection sensitivity. Oshana et al. (2020) also found that sexuality-related rejection sensitivity and sexual orientation concealment were predictors of BDD among 268 sexual minority adolescent boys and adult men (mean age = 24.6 years).

There is limited quantitative research on BDD in the context of physically intimate partner relationships. Survey research by Grant, Lust, and Chamberlain across a mixed-gender sample of mostly single 3,459 US students with a modal age range of 21–24 years, found that participants with BDD were significantly more likely than other participants to endorse compulsive sexual behaviours and substance-related impulsive behaviours. This participant group also showed greater comorbidity with PTSD, anxiety, and depression than other participants. However, it is difficult perhaps to establish from these findings the direction of relationships, or to achieve a contextual understanding of the mechanisms at play, including for example, any potential differences between genders.

## Female body image, sexual satisfaction, and self-objectification

Epidemiological studies suggest that BDD can often manifest differently in men and women, with men’s concerns more likely to revolve around hair thinning, nose, ears, genitals, and body build (i.e., “muscle dysmorphia,” Pope Jr et al., 1997), while women’s concerns are more likely to revolve around the skin of the face, the breasts, nose, and stomach (Phillips et al., 2006). Additionally, some research in appearance-related concerns has focused particularly on women. For example, women’s perceptions of their appearance and body have been found to influence the frequency and quality of their sexual experiences (Cash et al., 2004; Yamamiya et al., 2006). Self-consciousness has also been found to negatively influence women’s sexual satisfaction and sensations (Calogero & Thompson, 2009; Cash et al., 2004; Wiederman, 2012).

Preoccupation with appearance, self-consciousness, body shame, embarrassment, and body surveillance among women have been linked to self-objectification theory. This theory proposes that women internalize cultural or societal objectified norms of the female body, leading them to objectify their body during sex, thus becoming self-conscious, inhibiting their capacity for sexual enjoyment (Calogero & Thompson, 2009), and potentially leading to avoidance of sex (La Rocque & Cioe, 2011). Self-objectification theory usefully casts sexual experience as not only physiological, but operating within relational, psychological, and sociocultural frameworks (Steer & Tiggemann, 2008).

Sanchez and Kiefer (2007) have also found that sexual satisfaction is restricted by body concerns among men and women. This may operate through cognitive mechanisms: being cognitively distracted rather than “in the moment” may leave an individual unable to attend to their bodily sensations (Dove & Wiederman, 2000; Steer & Tiggemann, 2008). If sexual experiences or experiences of physical intimacy mean that the individual living with BDD is seen, or potentially seen by their partner, this could lead to negative self-evaluative cognitions, and thus detract from the possibility of sexual satisfaction (Calogero & Thompson, 2009).

## Qualitative research

Qualitative studies of BDD experience have shown that interpersonal difficulties are central among the phenomena experienced. Some studies have suggested that BDD is likely to lead individuals not to form partner relationships. For example, Silver et al. (2010) explored how individuals with BDD viewed themselves, using thematic analysis and visual techniques among a mixed-gender sample. One theme that emerged related to disordered interpersonal relationships. Participants felt unable to form relationships due to fears they were perceived negatively by others and also referred to real or anticipated difficulties with physical intimacy, despite these issues not having been the original focus of the study.

In a subsequent study among a mixed-gender BDD sample, Silver and Farrants (2016) found that experiences of mirror-gazing had such a paralysing and controlling effect on participants’ everyday lives, that they felt unable to live with others or form intimate relationships, as this would interrupt this ritualistic behaviour. Participants also found it difficult to comprehend how others could see them as attractive, given their self-perceptions from their mirror-gazing. Looking in the mirror led participants to imagine what an intimate partner might see and was thus off-putting to forming a real intimate relationship a partner.

Brohede et al. (2016) examined experiences of BDD including in the context of healthcare services among a mixed-gender Swedish sample. Overarching concepts included feelings of “imprisonment” and “being restricted in life,” in the context of relationships with family, friends, and intimate partners. In relation to the latter, participants reported (1) a lack of time for partners due to ritualistic behaviour, (2) partners’ lack of understanding and patience, and (3) a feeling that partners would be better off without them. Sexual intimacy was hampered by difficulties with being naked, not wanting to be touched, a constant focus on appearance, feeling unattractive, and not wanting or liking intercourse.

## Aims of this study

Social and interpersonal concerns form a central part of BDD experience and its DSM-5 diagnosis, and quantitative research has highlighted the severity of interpersonal problems among those with BDD in domains such as social inhibition, non-assertiveness, rejection sensitivity, minority stress and sexual orientation concealment (Didie et al., 2012; Fang et al., 2011; Oshana et al., 2020; Webb et al., 2015). However, there is limited quantitative research on BDD in the context of physically intimate partner relationships. It is difficult from mixed-gender survey research on BDD and sexual behaviour (e.g., Grant et al., 2019) to achieve contextual understandings, for example, of gender experience and BDD.

Qualitative research literature to date suggests that BDD may lead to an avoidance of partner relationships and physical intimacy (Silver & Farrants, 2016; Silver et al., 2010), a sense of separation, confinement and inadequacy in current partner relationships, and difficulties with physical intimacy (Brohede et al., 2016). However, studies to date have been in the context of mixed-gender samples, and no studies have focused centrally on physical intimacy experience in the context of current partner relationships.

Given the lack of research exploring BDD, physical intimacy and current partner relationships, the literature on female body image, sexual satisfaction and self-objectification theory, and the evidence that BDD may manifest differently in different genders, this study focuses on how women who identify with body dysmorphia or BDD made sense of their lived experience of physical intimacy in the context of current partner relationships, and aims to add to the growing body of research on interpersonal concerns in BDD.

In its focus on the production of new knowledge in light of diverse theoretical paradigms including the diagnostic paradigm, this study adopts a dialectical pluralist framework (Goertzen, 2010). This framework proposes that tensions between diverse or competing theoretical positions should be embraced as a useful foundation for investigative enquiry, and that an inter-contextualist approach, occupying a space between psychology’s positivist empiricist foundations and more critical approaches, should be adopted for the formation of psychological knowledge.

Additionally, in its focus on idiographic sense-making of embodied experience, this study draws upon the conceptual framework of critical realism (Bhaskar, 1975). As such, it incorporates an explicit ontology denoting the embodied phenomena of BDD as material, and the mechanisms producing interpersonal relationships as real structures that are in a state of flux and bound by cultural and social influences. Theoretical and empirical knowledge is formed relative to the context of these structural influences, through researcher and participant reflections that might potentially elicit change.

Based on these conceptual frameworks, this study aimed to integrate the existing theoretical and empirical knowledge base to produce a research question that could be analysed through an interpretative phenomenological methodology (Interpretative Phenomenological Analysis, Smith et al., 2009). Pre-existing knowledge was set aside to the extent possible during the data collection and analysis stages, in order to enable experiential phenomena to emerge in their own terms, and interpretations to be inductively drawn from the experiential data. The potential for reintegration of interpretative findings with the existing knowledge base is subsequently discussed.

## Method

### Design

This study has used Interpretative Phenomenological Analysis (IPA, Smith et al., 2009) to explore how women who identify with body dysmorphia or BDD make sense of their lived experience of physical intimacy in the context of current partner relationships. IPA was chosen as a more suitable methodology than descriptive phenomenology or thematic analysis because of its specific focus on cognitions and on sense-making in the context of situated, embodied experience (e.g., Larkin et al., 2011).

### Participants and recruitment

A purposive sample of six female participants, who identified with body dysmorphia or BDD and were currently in physically intimate partner relationships, was recruited through the BDD Foundation and OCD Action social media platforms (Facebook and Twitter) in the UK (UK). Participants were aged between 21 and 33 years old and had been in their current relationship between eight months and seven years, with an average length of relationship of three years, one month. All participants were heterosexual and White British. Four participants had received a formal diagnosis of BDD.

This sample size was in line with guidance from (Smith et al., 2009) for a study in partial fulfilment of a professional doctorate, where n = 4–10 is recommended (p. 52). In line with the aims of the study, a female-only sample was recruited. In particular, given findings women and men may experience different manifestations of BDD (Phillips et al., 2006), a mixed-gender sample might have introduced two structurally different phenomenologies, thus potentially disrupting the phenomenological homogeneity required by IPA.

Finally, participants in a current relationship were sought in order to ensure experiences of physical intimacy could be recalled as vividly as possible, and to avoid further confound or heterogeneity via longer-term memory recollections that might introduce new elements.

### Procedure

An in-depth semi-structured interview schedule was developed through iterative discussions between the first author, second author (research supervisor) and the second research supervisor. The aim was to ensure interview questions were directed towards the phenomena of interest and in line with the research question, while remaining sufficiently open to allow new phenomena to emerge during the interview, in accordance with an IPA approach to data collection (e.g., Pietkiewicz & Smith, 2014).

The final interview schedule was comprised of five substantive questions that allowed for a natural progression to the phenomena of BDD and physical intimacy, while also gathering broader contextual data from participants about what it was like to live with BDD. Questions about physical intimacy included, for example, how participants experienced getting undressed, showering, sexual intimacy and being naked in front of their partner. Data was collected via audio-recorded individual interviews of between 50 and 70 minutes, which took place at Regent’s University London.

### Ethics

The study received ethical approval from the DPsych Ethics Committee at Regent’s University London on 21 March 2018. A briefing and informed consent procedure preceded the interviews. All participants provided their informed consent to participate in the study. Given the study involved a sensitive topic and a single interview, in addition to a debriefing after the interview, which included the contact details of relevant UK help organizations, participants were also offered further contact with the researcher and the option to read their transcript in case they had anything to add or wished for any material to be removed. However, no participants took up this opportunity.

Methodological literature references difficulties in recruiting participants for qualitative studies of BDD due to the potential embarrassment and secrecy associated with the phenomenon (e.g., Pratt, 2014). However, there was a rapid influx of volunteers for this study. Participants expressed their desire to raise awareness and to help others understand what it is like to live with BDD in a current partner relationship. Following the interviews, participants expressed that, while discussing their experiences had to some extent been uncomfortable, it had also been helpful and valuable to them. Some participants suggested that the interview was the first time they had been able to talk about their experience outside of their close family.

### Data analysis

The data analytic strategy was grounded in the guidelines for IPA (Smith et al., 2009). Given the descriptive phenomenological component of the methodology, the first author used various strategies prior to the data analysis to bracket off pre-existing assumptions, theories, or interpretations (e.g., Chan et al., 2013), in order to attend as fully as possible to participants’ own sense-making of their experience during the prior data collection stage, while recognizing that some degree of hermeneutic process was inevitable even at this stage. After the interviews, the first author recorded their initial impressions, ideas, associations, or interpretations in a reflexive diary, to keep these separate, while retaining them for review later on.

Audio-recorded interviews were first transcribed verbatim; the transcriptions highlighted any pauses, changes in tone or non-verbal interactions as well as words spoken by participant and researcher. The first author continued a reflexive diary, so that further impressions, ideas, associations, or interpretations could again be captured for further review and potentially use in the analysis later on (e.g., Dowling, 2006).

Interviews were subsequently analysed sequentially; each interview was treated as an individual case study initially. The first author read and re-read each transcript alongside the audio-recording in order to become familiarized with the text and to get a sense of the whole transcript. The left-hand margin of the transcript was used for descriptive, linguistic, and conceptual codes (Smith et al., 2009). The right-hand margin was subsequently used to identify emergent themes, which were then clustered together to produce a table of superordinate themes, sub-themes, and quotations for each participant.

This process was repeated for each participant. Subsequent cross-case analysis, exploring convergences and divergences in the thematic data across participants, generated a summary table of subordinate themes grouped under superordinate themes that captured the meaning of the whole.

### Quality procedures

The data analysis was carried out primarily by the first author. While IPA recognizes researchers’ own role in co-constructing data interpretations with participants (the “double hermeneutic,” Smith et al., 2009, p. 3), and thus validates the notion that different researchers will inevitably generate different interpretations, nevertheless strategies to ensure rigour, trustworthiness, credibility, reliability, and validity were deployed during the analysis process (Yardley, 2008).

The initial coding of transcripts was checked by the second author for the relationships between descriptive, linguistic, and interpretative codes, to ensure the first author’s interpretations were grounded in a thorough descriptive engagement with the data, and not driven by their own biases or assumptions, while equally not remaining solely at the descriptive level.

Each subsequent data analysis stage was reviewed by the second author, which led to further amendments and iterations. The first author also sought feedback on anonymized data analysis extracts from a second supervisor and from a peer researcher. Finally, the second author reviewed a full audit trail, at the end of the analysis, to check for quality and consistency across the analytic process.

## Results

## The shame in being seen 

Participants expressed extreme discomfort in their naked or semi-naked body being seen by their partner and complex emotional responses to this. Being seen involved at times a sense of unbearable, excruciating exposure. Participants seemed to want to exist without a body, suggesting a feeling of shame in connection to their bodies. However, the observation of their partner was a painful reminder their bodies existed. Through their partner’s viewing their bodies were objectified (“it”) in the spatial and visual field. Participants’ own judgement of their bodies seemed to find itself within their partners’ imagined viewpoint or appraisal; participants attempted to protect themselves from this painful appraisal by avoiding being seen. All participants are referred to by pseudonyms below.

### Sheltering from judgement

All but one participant spoke about the ways they prevented themselves from being seen naked or undressing by their partner, in order to protect (“shelter” Samantha) themselves from the judgement or shame they might experience under the observation of their partner. Participants covered their body with objects, makeup, or clothing, or sheltered behind the wardrobe or in the bathroom, both in order to hide and to be more appealing to their partners.

I’ll, like, take my clothes to the bathroom or I’ll do it behind the wardrobe […] I’ll always do it sheltered […] he never watches me get changed fully, or whatever, because […] I don’t want him to see it **(Samantha)**

Through protecting themselves, it seemed as if participants tried to protect their relationships by giving their partners a “sheltered” experience of intimacy, shielding them also from the unpleasant reality of their perceived flaws. Make-up was another form of cover, creating a “nice pattern on my face (Mia).” Without make-up, there seemed to be a further sense of shame or embarrassment, and hands became like the wardrobe door:

I do often, like, hide my face when I don’t have my make-up on. I’ll just be like, “You can’t look at me [behind hands in the interview]” **(Mia)**

While these experiences may seem commonplace or a matter of preference rather than necessarily indicative of BDD per se, the unique factor seemed to be the level of distress that accompanied these experiences, and the way these experiences formed part of a wider set of distressing phenomena. In parallel, this distress seemed to manifest in the way participants recalled their experiences during the interviews—some participants were tearful, others seemed to hold a tense or hunched posture, or to look down, or to express the need to take a break, as if in the recall of experience, some shelter was also needed.

### Controlling and manoeuvring the gaze

All participants attempted to control and manoeuvre their partner’s gaze as a means of managing their discomfort during physical intimate times when they could not avoid being naked or exposed, such as during showering or sex. Participants moved their naked bodies into certain positions or particular angles to help control their partners’ gaze and avoid feeling vulnerable or exposed.

I don’t want him to see it in the shower. I don’t let him see me change, so I’m definitely not gonna let him see me fully just stood in one position, ‘cos then he can really look at me […] I would probably not really be showering, because I’d be trying to work out what way to stand to look best … and appealing **(Samantha)**

The participant’s concern was that in the shower her partner could “really look” at and see the “real” “it.” A second participant used “it” when describing walking backwards in order to create certain angles, perhaps acting as if she wished to be in two dimensions rather than three:

I’m, not comfortable in it, there’s definitely certain angles I wouldn’t want him to see me from […] I’d probably walk backwards [laughs] so he doesn’t have to see my bum, just make a bit of a joke of it **(Mia)**

For this participant, making “a bit of a joke” might be a way to diffuse difficult feelings—laughter might be another form of control, manoeuvre, or camouflage, an attempt to hide a difficult and painful experience, and prevent this from being exposed. Again, during these parts of the interviews, the intense way that participants recalled these experiences (with laughter, repetition, pauses, turning away) was felt to be indicative of the importance of these manoeuvres for them, and the degree of distress these manoeuvres were perhaps helping participants to avoid.

### Public versus private

Around half the participants suggested there was a difference between the gaze of a stranger and their partner’s gaze. One participant, who had experienced BDD during a period in her life when she worked as an exotic dancer, found that dancing in front of the “blokes” who used to watch her was helpful to her body dysmorphia. The exotic dancing seemed to imply the possibility of difference, both in the bodies of the dancers, and in the preferences of the audience. In contrast, being seen within the emotionally bound and more singular context of an intimate relationship (“my other half”) was harder to accept:

I was an exotic dancer for a bit, and I, when I was doing that, it’s so weird, it helped my BDD, because I was working with girls that were all different shapes and sizes, … you know that blokes like all different things, and don’t really care about stuff, when it comes to being with, with my other half, I think, ‘Oh, he’s … ‘ I’m more critical of myself **(Victoria)**

In contrast, another participant suggested that the longevity of her relationship and the fact she had habituated her partner to all her “flaws” made the intimate relational context more comfortable than the public exposure of a changing room, beach, or swimming pool. It seemed as if the intimacy with her partner perhaps allowed her not to see her body as an “it” but that this became exposed in a public place:

I don’t really have a problem with that […] we’ve been together for so long […] I’m so comfortable with him that all my flaws […] I’ve already drawn so much attention to them […] I’ll be really self-conscious in a public changing room about people seeing my body … like on the beach or in the swimming pool, or anything like that **(Bethany)**

It is interesting to note perhaps the dynamic relationship between intrapersonal experiences of BDD and the interpersonal environment, which may equally be subject to diverse social or cultural norms, for instance, here, in relation to the normalcy or not of nakedness in public changing rooms, beaches or swimming pools.

## Disgust and detachment during intimacy

Participants described disgust and a sense of detachment or disembodiment during physically intimate moments with their partners. The intimacy was uncomfortable and exposing—embodying an objectified sense of self as “deformed” (Bethany, Lucy) “ugly” (Grace, Mia, Samantha) “gross” (Grace, Samantha) and/or “fat” (Bethany, Grace, Mia, Samantha, Victoria), participants struggled to be intimately “in the moment”. In speaking about their experience during the interview, participants again seemed to disconnect from identifying their body as their own, referring to their bodies as “your body” or “it” (Bethany, Grace, Mia). The experience of physical intimacy was accompanied by a cognitive focus on the appearance of their body and highly negative appraisals of its appearance.

### “I’m just outside watching”: Disembodied being

Unable to cope with physical intimacy, participants expressed separating from their physical body. Detaching from their bodies seemed to shield them and help them escape from a potentially threatening situation:

I think I definitely have like a feeling of just being detached … I’m not really like present in it … I always feel like I’m just outside, watching **(Bethany)**

All participants engaged with a critical internal dialogue, adopting a third-person perspective through which they monitored and evaluated their bodies. Their preoccupied thoughts and worries ranged over their bodies as a visual stimulus, and took at least half of their brain away from a role as an active participant, being in the present moment:

worrying in some way what I appear like or what I’m doing or what he’s seeing or what he’s looking at, erm, definitely, as opposed to just kind of, I guess, be in the moment […] half your brain is preoccupied with …, ‘Can he see my face? Is my face being ugly? … what my hair’s doing, erm …, “Why am I bending over like this?” or my fat’s like bunching together **(Mia)**

One participant described seeing an array of images of the bodies of “beautiful women” being played out like a “slideshow” in her mind when having sex. There was a sense of disembodied, cut off experience, while at the same time a visceral experience of confusion. Making negative comparisons between her own body and the moving sequence of images of other girls in her mind was so upsetting that she was frequently unable to continue. Like the previous participant, who questioned what she looked like and how she was positioning her body, this participant was also led to question herself, perhaps to question whether it was feasible to engage in physical intimacy at all:

if we are having sex […] I see pictures in my head of like other girls and like just sort of, just sort of start thinking, […] just feel like gross, […] in my head it’s like a slideshow of bodies, beautiful women that I don’t look like. And then I just feel really bad and … just feel like … disgusting [laugh] … A lot of the time I end up like stopping it … because I’ll either start crying or I think “What am I doing?” **(Grace)**

In participants’ narratives, BDD seemed to take on an identity of its own—as though there were three in a relationship, one of whom was a critical or superior observer sabotaging, controlling, or restricting their freedom during physical intimacy, and making participants act in certain ways. BDD was a bully or abuser—one participant suggested that like a bird it “pecks at you” in a way that sounded as if it might feel like a physical pain. BDD’s negative self-talk hounded her with its constant, sharp jabs:

I guess kind of ruins it when you’re trying to be intimate. Like the body dysmorphia has its little … [sigh] thing to everything, its input to everything, […] it doesn’t give you peace. It just kind of pecks at you **(Lucy)**

### “Waiting for it to be over”: Struggling for pleasure

Most participants struggled to have any sense of pleasure in the act of intimacy; rather it was a numb experience, disengaged from their bodily senses.

I can’t really feel it. Like I can, OK, like I know there’s a sensation happening, but I get no like good [sniffs] feelings from it or anything **(Lucy)**

Sex was described as a mundane obligation (a “chore just to get it over, over and done with” Lucy), something to get through for their partner’s pleasure, an ordeal (“test” Mia) or a functional task (“goal-orientated” Mia) rather than a relational or pleasurable activity.

Some participants referred to sex as “performance” (Grace, Mia) influenced by internalized messages about how sex should be with a pressure to have an orgasm the marker of successful sex. Others seemed to perceive the performance more in terms of being staged (“are you making the right noises” Mia) to give the impression of being sexually aroused and in the moment.

Another participant’s narrative suggested anxiety got in the way of other feelings—perhaps sex felt threatening and unsafe. They described going into a state of freeze as if disconnecting from the process:

I’ll just freeze up, trying to act in a way that … I know I should be, by kissing and foreplay, but I’m just not feeling it, I just can’t wait for it to be over, it’s kind of a performance **(Grace)**

Finally, many participants had a negative physiological response to their partners touch and experienced the need to get out, or a feeling of being trapped inside an unwanted body. Beyond the lack of pleasure, sex became a physically distressing experience involving nausea and disgust:

Skin’s like crawling and I just wanna like get out of my body (…) I start to like feel a bit like nauseous and … yeah, just feel a bit like sick **(Grace)**

## A flawed self unworthy of relationships

All participants expressed being unworthy of being in a relationship due to their appearance. Most participants questioned why their partners were with them and wondered how their partners could have been deceived. Participants saw themselves differently to how they thought their partners saw them, as if their partners were yet to see them the way they really were. These self-perceptions and references to partners’ perceptions were oriented around appearance and did not refer to self attributes beyond physical attributes.

### “What if he saw me for who I was”: The imposter syndrome

Participants suggested their partners’ desire to be physically intimate with them was only present because those partners were yet to see their real selves, which were flawed and undesirable. Participants seemed to live with a fear that if their partners were to see them in the way they “really” are, they would abandon them (“what if”). It was as if they were living in a secret world that might one day be found out:

what if he saw me for who I, how I really was, like … what if one day he just woke up and seeing all the flaws that I saw **(Lucy)**

One participant’s partner was partially blind and had recently been offered corrective eye surgery. She envisioned his regaining of his vision as if suddenly the world would look different for both of them; it would lead him to see her in sharper focus or see parts of her he had not seen before. There was a sense of sadness in the interview as she anticipated the potential loss of her partner (“Oh wow”); her lowered head during the interview suggested she might be experiencing or anticipating experiencing shame:

I worry like he’d eventually … He’d get it back [lowered head], and I just get this whole vision of, “Oh wow, like he didn’t see me as in detailed,” or, he would see something that he didn’t before **(Samantha)**

### “They couldn’t have liked me for me”: Feeling worthless and undeserving of intimacy

At points participants’ narratives seemed to suggest disbelief that they had any value or worth for their partner. One participant expressed feeling so unworthy of her partner that by not being physically intimate with him, she was protecting him from catching her deformity:

It kind of felt like, if he touched me, he, he’d like catch deformity […] I’d rub off on him […] I’d impact him in a negative way **(Lucy)**

Participants expressed feeling inadequate (Samantha, Mia, Grace, Lucy), believing they were “not good enough” or “not beautiful enough” (Lucy, Mia, Grace, Victoria, Samantha). One participant felt inferior to her partner and doubted why he was with her, given his “muscular” physique and because he was an “attractive person” (Victoria). Similar negative self-appraisals and upward comparisons have left participants feeling undeserving of sexual enjoyment:

I don’t feel like I deserve to be touched like that ‘cos I’m not good enough (…) I wasn’t worthy of … enjoying sex **(Lucy)**

Many participants found it hard to comprehend why their partners found particular parts of their body desirable, almost as if it was a joke. It seemed there might be a link between an inadequate sense of self and inadequate parts of their body, which they described in terms such as “awful,” (Mia) “giant” (Samantha, Grace) or “rubbish” (Samantha). There seemed to lead to a disruption of the self—one participant seemed to struggle to locate themselves in the situation (“you” … “me”):

How is he finding … that bit of my body sexy?’ Like when he touches my bum, I’m like, ‘Seriously, it’s giant […] “How are you finding that flabby bit of bum, sexy?” like […] boobs—I’m just like, well, there’s nothing of them and they’re just rubbish and … You just feel like inadequate … when he touches me **(Samantha)**

Participants also expressed doubt as to why their partners were with them. For one participant, this doubt led to a suspiciousness—there was something odd that they did not understand. Some potentially more acute or paranoid thoughts about their partner being misled or coerced seemed to have arisen. However, it seemed somewhat slippery or unclear to the participant who was doing the coercion (“something else” … “somebody” … “I was doing him an injustice”).

being paid to go out with me […] they couldn’t have liked me for me. […] “There’s got to be something else here,” or has somebody threatened them to go out with me? […] I felt like I was doing him an injustice by him being with me **(Lucy)**

## Discussion

This qualitative study has aimed to provide a detailed contextualized account of how women living with BDD make sense of physical intimacy within current partner relationships, in order to take previous qualitative research literature forward, and to contribute to the growing body of BDD research focusing on interpersonal concerns. Its findings are broadly in line with quantitative research that highlights the place of social inhibition, non-assertiveness, and rejection sensitivity in BDD (Didie et al., 2012; Fang et al., 2011; Oshana et al., 2020; Webb et al., 2015), while illuminating how these variables might play out in the context of current partner relationships.

### Shame mirrored in the other’s gaze and mind

Previous qualitative research highlighted BDD participants’ avoidance of partner relationships and physical intimacy (Silver & Farrants, 2016; Silver et al., 2010), and feelings of separation, confinement, and inadequacy, as well as difficulties with physical intimacy (Brohede et al., 2016). In line with these findings, this study’s participants expressed immense fear and difficulty in being seen naked or partially naked, in being touched and in having intercourse. However additionally, and consistent with quantitative research suggesting that shame is a correlate of BDD (e.g., Sündermann et al., 2016), this study highlights the central place of shame, rooted in being subject to the gaze of a partner. Participants expressed that in being seen, they felt exposed, vulnerable to their partner’s judgement, and ashamed of their bodies.

In comparison with Silver and Farrants’ (2016) study of mirror-gazing and BDD, the gaze of the partner in this study was perhaps acting like a kind of mirror, through which participants tried to make sense of their appearance by imagining what their partner might see. Participants struggled to comprehend why their partners were with them and for some this was because their partners were yet to see the “real” version of themselves, just as of course the mirror does not. Similarly, Silver et al. (2010) found that participants felt unable to form relationships in the first place in case their real, defective self would be exposed, suggesting these dynamics are at play both in avoiding and in engaging in physically intimate relationships.

Veale’s cognitive-behavioural model of BDD (Cash et al., 2004) suggests that when individuals are confronted with an external representation of their appearance (e.g., mirrors or photographs), a distressing negative cognitive self-appraisal is triggered. This study suggests the scope for triggers of distress can be extended into the context of an intersubjective relationship, in which those living with BDD are subject to an imagined image or representation in a partner’s mind. This suggests that being in an intimate relationship may represent a repeated trigger for the cycles of distressing thoughts, feelings, and actions proposed by the cognitive-behavioural model of BDD.

Participants’ attempts not to be visible or not to be seen—to find shelter behind objects or to control or manoeuvre a partner’s gaze—can be psychologically understood as ways of defending or coping with their intense distress and feelings of shame. These attempts to shelter, control, or manoeuvre are also in line with Veale’s cognitive behaviour model (Cash et al., 2004), which proposes that individuals with BDD use “safety behaviours” in an attempt to manage their distress. From a cognitive-behavioural perspective, these safety behaviours are self-defeating, as they do not allow for opportunities to experience the feared situation and thus survive its consequences.

These findings relating to shame and attempts to cope with shame by hiding are also clearly reminiscent of existential phenomenological theories, such as Fuchs (2002) who has suggested BDD involves an embodied phenomenology of shame that operates through the gaze of the Other, akin to Sartre’s phenomenology of the body and wish to be invisible (“not to have a body anymore,” Sartre, 1969, p. 353) Like it or not, the body is made visible by the other’s gaze. The body-subject becomes a body-object or body-for-others (Merleau-Ponty, 1962/2002), visible to others and therefore susceptible to evaluation and critique (Fuchs, 2002). Participants’ belief their partners may not know their real selves may link to a disembodiment experience in BDD (Mitchell, 2017; Morris, 2003) arising from the difficulties for the lived body under conditions in which it is observed by others (Fuchs, 2002).

While their partner’s gaze may have acted like a mirror, the images experienced were of participants’ own making, just as the image seen in the mirror is seen by our own eyes and does not come from the mind or sight of the mirror. From a psychoanalytic object relations perspective, participants can be seen as attempting to make sense of their body-self through the purview of their desired object (Lemma, 2009). In line with mentalization theory, a contemporary development of object relations theory, it could be argued this situation involves a difficulty in mentalization (Fonagy et al., 2018), that is, in conceiving of one’s own and other people’s minds as separate, different, and ultimately unknowable or opaque (Fonagy & Target, 1997).

For participants in this study, the visions in the minds of their partners remained out of their awareness, and perhaps this fact remained out of their awareness also. Instead, the images they described were a playback of their own image-making activity. It could also be argued that an unconscious projective process (Klein, 1946) was at play in which participants’ attempts to defend themselves involved placing their minds (thoughts, images) in their partner’s mind. The ensuing fusion of minds does not allow for a separation between minds, and in these circumstances, it would understandably be difficult for participants to understand why their partners liked them or wanted to be with them. Overall, these experiences seemed both to be contextualized by and to contribute to deeper feelings of worthlessness and low self-esteem. For some participants, there was anticipated rejection and sadness that they might lose their partners; for others, disbelief that their partners found their bodies attractive almost as if it were joke; one participant seemed suspicious as to who or what had led their partner to stay.

### Trauma, dissociation, and objectification

Experiences of detachment and disembodiment during physical intimacy led to difficulties with being in the moment or experiencing pleasure or enjoyment for participants; instead, their feelings included anxiety, nausea, and disgust. Participants’ detachment and disembodiment are indicative of dissociation from the lived experience of intimacy (“I’m just outside, watching”). Linked to the possibility that being in a physically intimate relationship might lend itself to frequent triggers of distress, the notion of dissociation is often associated with trauma (e.g., Hoeboer et al., 2020). There could be an element of repetitive trauma involved in being in a physically intimate relationship while living with BDD. If sexual intimacy acts as a trigger, some participants responded to this by interpreting sex as unsafe and detaching through a freeze response.

Other participants characterized sexual intimacy as functional or something to endure, or as performative in the sense of staged. Physical intimacy with their partners became a (stage) “act to be done.” This highlights a move within BDD experience away from sex as relational and towards an act to be done (to), rather than an experienced (with) another person. This performative response could be seen as another form of dissociation in the context of traumatic self-other experience (e.g., Schimmenti & Caretti, 2016).

Another possible indication of trauma is participants’ experiences of images of themselves and of other women in their mind, during sex, which detracted from the intimacy experience to the extent that they either longed for it to be over or were unable to continue. These images could be seen as intrusive images and may thus be evidence of the presence of trauma. This finding would support the findings of Osman et al. (Goertzen, 2010)’s study in which BDD patients reported spontaneously occurring appearance‐related images that were significantly more vivid, detailed, negative, recurrent, and viewed from an observer perspective than images of control participants.

Links between BDD, dissociation and trauma are documented in the quantitative literature. For example, Dyl et al. (2006) found that among an adolescent psychiatric sample, participants with body shape or weight concerns had significantly greater symptoms of PTSD, dissociation, and sexual preoccupation or distress. Similarly, Sündermann and Veale (2017) suggest complex BDD in adults is associated with dissociative experience and trauma. Möllmann et al. (2019) found that gazing at facial features increased dissociation among a non-clinical female sample, suggesting a self-perpetuating cycle of triggers of distress and attempts to cope with it.

Participants’ critical thoughts about their body object from an observer perspective appeared to be both a source of distress and a means through which to disengage or dissociate from distress. Participants placed significantly more importance on their physical appearance as perceived by their partners (their body-object), than on their own subjective felt or internal sense of self (their body-subject). In this context, in line with self-objectification theory, participants experienced their bodies as objective images (Fredrickson & Roberts, 1997). This self-objectification, operating through cognitive distraction, led to difficulties being in the moment, and reduced enjoyment derived from sex (Calogero & Thompson, 2009; Dove & Wiederman, 2000; Steer & Tiggemann, 2008; Wiederman, 2012; Yamamiya et al., 2006).

### Limitations of this study

The key limitation of this study is that its scope was restricted to a small sample of women and did not include men’s experience, nor a broader range of participants in terms of past and current relationship status. Additionally, while this was not a sampling criterion, all participants were cisgender. This study has not considered the comparative literature around transgender experience of body dissatisfaction and dysmorphia (e.g., Namla et al., 2019).

Furthermore, all the women in this experience identified as heterosexual. Research has suggested that all women experience dissatisfaction with their physical appearance and societal pressures irrespective of their sexual orientation (Heffernan, 1996; Peplau et al., 2009; Share & Mintz, 2002). This study has only captured the experience of one particular group of women, and it is unclear from this study what divergences or convergences might be found within the meaning-making of physical intimacy experience among women of other sexual orientations.

A final limitation is that all participants identified as British Caucasian. As such this study has not explored body dysmorphia and physical intimacy experience among a greater diversity of ethnicity or culture, which might entail important differences (e.g., Dixon & Marques, 2017).

### Clinical implications

Clinical pathways for adult BDD interventions in the UK involve a stepped care approach (National Institute for Health and Care Excellence, 2005). Individuals with BDD with mild functional impairment are offered cognitive-behavioural therapy (CBT) including exposure and response prevention (ERP); with moderate functional impairment, a choice of either an SSRI or more intensive individual CBT (with ERP); and with severe functional impairment, combined SSRI and CBT (including ERP) interventions.

This study indicates the potential overlap between BDD and everyday concerns people may have about their appearance, while also differentiating BDD from such concerns in the degree of distress that accompanies the experience. Assessing the source, function and impact of appearance concerns is relevant to clinical decision-making in cosmetic, surgical, or dermatological treatments (e.g., Dufresne Jr et al., 2001). NICE guidelines (2005) state that for people with mild disfigurements or blemishes seeking aesthetic procedures, healthcare professionals should routinely consider the possibility of BDD, and that those with suspected or diagnosed BDD seeking aesthetic treatment should also be assessed by a mental health professional with expertise in BDD. Medical ethics guidelines recommend that while patients’ aesthetic concerns should not be dismissed, aesthetic intervention is to be carefully considered against the risk of failure to meet the patient’s expectations or to address the underlying issues in the absence of accompanying psychological intervention (e.g., Lane, 2020).

This study lends weight to the importance of understanding BDD as a psychological phenomenon, and has implications for counselling psychologists, psychotherapists, and other mental health practitioners working with BDD. NICE guidelines (2005) recommend that psychological assessment should cover interpersonal functioning at work and within friendships. In line with Sündermann and Veale (2017), this study highlights the importance of a full functional assessment of the interpersonal correlates, as well as the intrapersonal correlates, of BDD experience. This assessment could also include sensitive questioning about the impact of BDD on partner relationships and vice versa, and thus open a space for a potential exploration of the place of physical intimacy among the concerns, where this is felt to be relevant. Such an assessment would feed into an idiographic contextualized psychological formulation as the foundation for subsequent intervention (e.g., Johnstone & Dallos, 2013).

In the context of psychological intervention, this study is informative of the wide range of cognitions, behaviours and emotions associated with BDD in the context of intimate partnerships, which may include fear, anxiety, paranoia, shame, disgust, trauma, and dissociative experience. These phenomena may also appear in the therapy room within the relationship between client and therapist. Practitioners must work sensitively with potential parallel processes, holding in mind the potentially protective function of disengagement and dissociation in this context, and thus the importance of asking for the patient’s perspective on the areas they would like to work on, without making assumptions.

In light of these findings, mental health practitioners may wish to consider adjunct interventions to CBT/ERP such as compassion-focused therapy (Gilbert, 2011; Veale & Gilbert, 2014), to build clients’ ability to self-sooth and reduce feelings of shame. Imagery rescripting (Arntz, 2011; Ritter & Stangier, 2016) may also be considered a useful addition to CBT/ERP, to work with spontaneously occurring images that may form a central part of traumatic experience. If clients wish to work with connecting with their body-subject (their lived body), interventions such as mindfulness-based cognitive therapy (Segal et al., 2018), movement-based therapy (Keating, 2020), or focusing (Gendlin, 1969) may be considered.

### Future research

This research is concerned with the intersection between intrapersonal experience and an intersubjective space—that of being physically intimate—for people living with BDD. Future research could explore how partners experience physical intimacy with someone who is living with BDD, allowing a multi-perspectival understanding of this intersubjective phenomenon (Larkin et al., 2019). There would be value in such a study to support practitioners working with BDD both with individuals and in the context of couples’ therapy.

Previous research has suggested there are differences between women and men’s experience of BDD (Krebs et al., 2017; Phillips et al., 2006, 2010). While this study focused on women’s experience, there would be value in future research exploring how men with BDD make sense of physical intimacy in current partner relationships, to further support assessment, formulation, and intervention in clinical practice. Future research exploring differences between or within diverse genders, sexualities, ethnicities, or cultures in relation to BDD and physical intimacy would also be beneficial.

## Conclusion

This study has explored how women make sense of living with BDD in the context of physically intimate partnerships and demonstrated how appearance-related concerns filter into the cognitive, behavioural, and emotional intersubjective spaces of these relationships. Shame and trauma experiences may be triggered by being observed or by nakedness, touch, or intercourse, and may be managed or coped with through disengagement or dissociation.

It is hoped this study will contribute to the body of research on interpersonal phenomena in BDD and support mental health practitioners in working with this painful issue. Participants’ expression of the value in participating in this study also validates the need to continue to overcome recruitment challenges and conduct further research, both to support those living with BDD, and the practitioners whose aim is to help them.

## Key points of this study

- This study highlights difficulties around nakedness, touch, and intercourse for women with BDD.
- Participants experienced shame, trauma, and dissociation in response to physical intimacy.
- Compassion-focused therapy, imagery rescripting or mindfulness-based approaches may be helpful clinically.
- Future research among partners of those living with BDD, and among diverse genders, sexualities, ethnicities, or cultures is recommended.
